# Using deep vision-language models improves multi-task performance in assistance applications for endoscopic ENT surgery

**DOI:** 10.1007/s11548-025-03512-z

**Published:** 2025-12-22

**Authors:** Richard Bieck, Martin Sorge, Katharina Heuermann, Viktor Kunz, Markus Pirlich, Thomas Neumuth

**Affiliations:** 1 Innovation Center Computer Assisted Surgery (ICCAS), Leipzig University, Semmelweisstraße 14, 04103 Leipzig, Germany; 2 Department for Ear-, Nose- and Throat- Surgery, University of Leipzig Medical Center, Leipzig, Germany

**Keywords:** Image-based endoscopic navigation, Deep learning, FESS, Vision-language models, Pre-training, Image embedding, Text embedding, Transformers, Explainability

## Abstract

**Purpose:**

Deep learning models for endoscopic assistance applications predominantly focus on image-based tasks, such as tool detection, anatomical classification, and workflow segmentation. However, these approaches often neglect the integration of natural language, limiting their assistance capabilities. This work adopts a proven architecture for vision-language models (VLM) to perform multi-task learning for image classification, text prediction, and surgical report generation, specifically for endoscopic ENT surgeries.

**Methods:**

We adopted a VLM architecture utilizing encoders biased for the endoscopy domain for image and text embedding and combine them via cross-attention. The model was trained on a newly created multi-task dataset derived from 30 annotated endoscopic procedures, comprising 130,000 multi-label images, anatomical descriptions, and synchronized surgical reports. Two variations of the model, a lightweight 61M parameter and a 176M parameter model, were evaluated both against an existing baseline from previous mono-task studies as well as the EndoVit and SurgicalGPT models as external references. Ablation studies investigate the influence of removing image or text embeddings, and cross-attention on the task performance. Performance was measured for landmark classification, structured text prediction, and report generation using precision, recall, *F*1-score, BLEU-2, ROUGE-L, and cosine similarity metrics.

**Results:**

The VLM base model improves the baseline *f*1 score for image classification by up to 12% and natural language text generation by up to 14% across image classification and report generation tasks. The text generation of structured language tasks, however, showed minimal gains, indicating limitations in structured sentence learning from combined image-text embeddings. EndoViT and SurgicalGPT slightly trail our domain-specific VLM. The image-only and text-only ablations confirm that the vision component benefits language tasks, whereas text has limited impact on landmark detection.

**Conclusion:**

We developed a vision-language model capable of integrating image and text data for endoscopic ENT assistance tasks, that is able to replace three isolated models, delivering multi-task assistance while outperforming prior and general‑purpose baselines. Remaining challenges include the handling of imbalanced class distributions and limited gains on templated structured text.

## Introduction

Endoscopic assistance systems have traditionally relied on vision-only deep-learning pipelines, e.g., detecting surgical tools, segmenting anatomy, or recognizing workflow phases [4] directly from video frames [5]. While these single-modality models achieve high accuracy, they lack the ability to reason over the rich textual context that accompanies a procedure, such as anatomical descriptions, surgeon commentary, or structured report fields.

Recent studies show that coupling visual encoders with language decoders enables tasks such as question answering [1], captioning and zero-shot classification across gastrointestinal and laparoscopic scenes [6, 11]. These works demonstrate that large-scale pre-training on image-text data can transfer to endoscopy with minimal fine-tuning and that semantic embeddings help tackle long-tail class distributions and unseen tool categories. They also underscore a rapid shift toward multimodal understanding in endoscopy. 

Despite this progress, multi-task assistance in ENT sinus surgery remains largely unexplored. Moreover, large language and multimodal models add too many constraints for a potential real-time scenario in the operating room.

We, therefore, adapt a compact VLM architecture for endoscopic ENT assistance that jointly handles three complementary tasks: (i) multi-label anatomical-landmark classification, (ii) prediction of the next anatomical description, and (iii) generation of the next surgical-report sentence. By coupling a vision encoder with a lightweight language decoder via cross-attention, our model injects visual context into language prediction while keeping computational cost modest. Our key contributions are:

1. We show that one compact multi-task VLM architecture can simultaneously handle visual classification and two text-generation tasks, providing a compact pipeline instead of isolated model instances.
2. We present a curated multimodal dataset that aligns video frames with synchronized landmark labels and time-stamped surgical report sentences, offering a new resource for multimodal learning in sinus procedures.
3. Through external baselines, ablations on modality fusion, and class-wise errors, we quantify how cross-modal information improves task performance and identify remaining challenges such as long-tail imbalance and structured-text limitations.

We are focusing on the sinus-surgery domain, where our earlier work on single-task assistance functions can now be unified inside one compact vision-language model. Leveraging the same annotated video corpus guarantees us comparability to prior work and lets us quantify the added value of cross-modal fusion while avoiding costly new data collection. Moreover, ENT sinus surgery offers a confined but visually complex workspace that stresses inference and small-footprint deployment, making it ideal for compact, task-integrated multimodal models.

## Related work

Vision-language models (VLMs) have demonstrated exceptional potential across diverse applications by aligning image and text representations, particularly in tasks like generation, classification, and retrieval. In medical imaging, systems like MedFuseNet [1] have achieved state-of-the-art performance in visual question answering (VQA) by effectively integrating attention mechanisms to focus on relevant regions and textual elements. Compact architectures, such as those in [2] and [3], have shown that resource-efficient models, when optimized with high-quality annotations, can perform on par with larger ones, making them suitable for computationally constrained environments like operating rooms.

In endoscopic applications, vision-based navigation systems [4] have automated guidance through anatomical classification and positional inference, while holistic surgical scene understanding [5] has demonstrated the value of integrating visual and textual data to improve workflow comprehension. Natural language processing (NLP) approaches for surgical report generation [6] have reduced documentation burdens, leveraging structured and free-text inputs to streamline reporting. Recent years have seen a surge of vision-language research in endoscopy. SurgicalGPT [7] extends GPT-style decoders to accept visual tokens and achieves top-ranked scores on the MEDVQA-GI 2023 benchmark for laparoscopic visual–question answering. EndoViT [8], a ViT backbone pre-trained on 18,000 endoscopic videos, has proven highly transferable across polyp detection, phase recognition, and report generation tasks. Foundation-model initiatives such as NasVLM [9] for nasal endoscopy and EndoVLA [10] for robotic tracking further illustrate the field’s rapid expansion. Recently, Märkl et al. proposed an VLM approach for zero-shot multi-label endoscopic instrument classification [11], demonstrating that sentence embeddings can act as class prototypes to substantially boost zero-shot recognition of surgical tools in cholecystoscopy images. Their work, while focused on instrument detection rather than anatomical assistance, further illustrates the rapid progress of multimodal and semantic-embedding approaches in endoscopy. Compared with these works, our study targets ENT sinus surgery and unifies three assistance tasks in one compact architecture, complementing the large-scale, often single-task focus of existing models.

While two of the tasks are essentially text-generation problems, they are inherently grounded in the visual scene and are dependent on labeling for accuracy. The current endoscopic scene constrains which anatomical region can plausibly be reached next and determines which report phrases are clinically appropriate. Using a VLM, we are including image-conditioned bias that would not be possible with pure language models. By integrating previously uncoupled datasets, we produce text and image embeddings to improve overall task performance. We build on the success of prior VLMs and adopt their application in a comprehensive multi-task framework specifically tailored for endoscopic ENT surgery, addressing unique challenges for the surgical domain, such as aligning datasets, annotation consistency, and computational constraints.

## Data and tasks

This work introduces our adoption of a proven vision-language model (VLM) architecture that we customized for multi-task deep learning in endoscopic ENT surgery. Data were collected and annotated to align sequential surgical artifacts, including endoscopic frames, surgical keywords, and report sentences, into a vision-language dataset. The VLM employs an encoder-decoder structure, leveraging pre-trained and fine-tuned encoders. For the multi-task dataset, three different task-specific datasets were combined. We previously trained deep learning models on subsets of data from the same database of functional endoscopic sinus surgery (FESS) procedures and extended them to now cover the same video database (*n* = 30) consisting of 16 h of ENT procedures. The result was a dataset, for which each datapoint included an associated multi-label class (task 1), a current and future anatomical description (task 2) as well as the most recent part of a surgical report and a keyword (task 3). In the following sections, the specific tasks and task losses are described in detail followed by details on the combined dataset.

### Multi-task dataset preparation and statistics

**Fig. 1:**
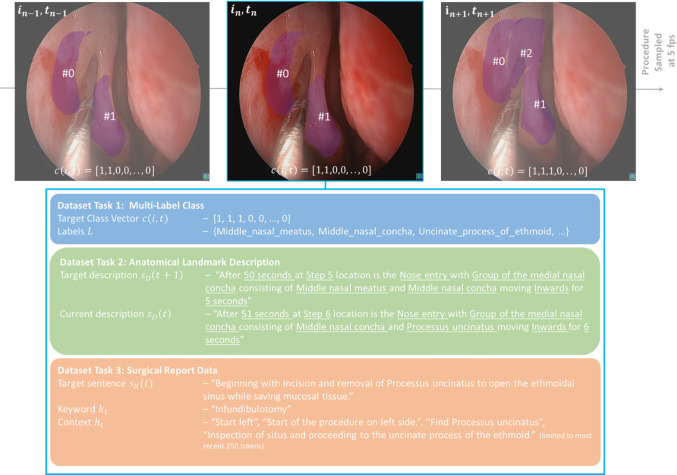
Overview of a data point in the multi-task dataset. Task-specific data is differently colored. Anatomical landmarks are additionally indicated as regions-of-interest in endoscopic images and are not part of the dataset

**Fig. 2:**
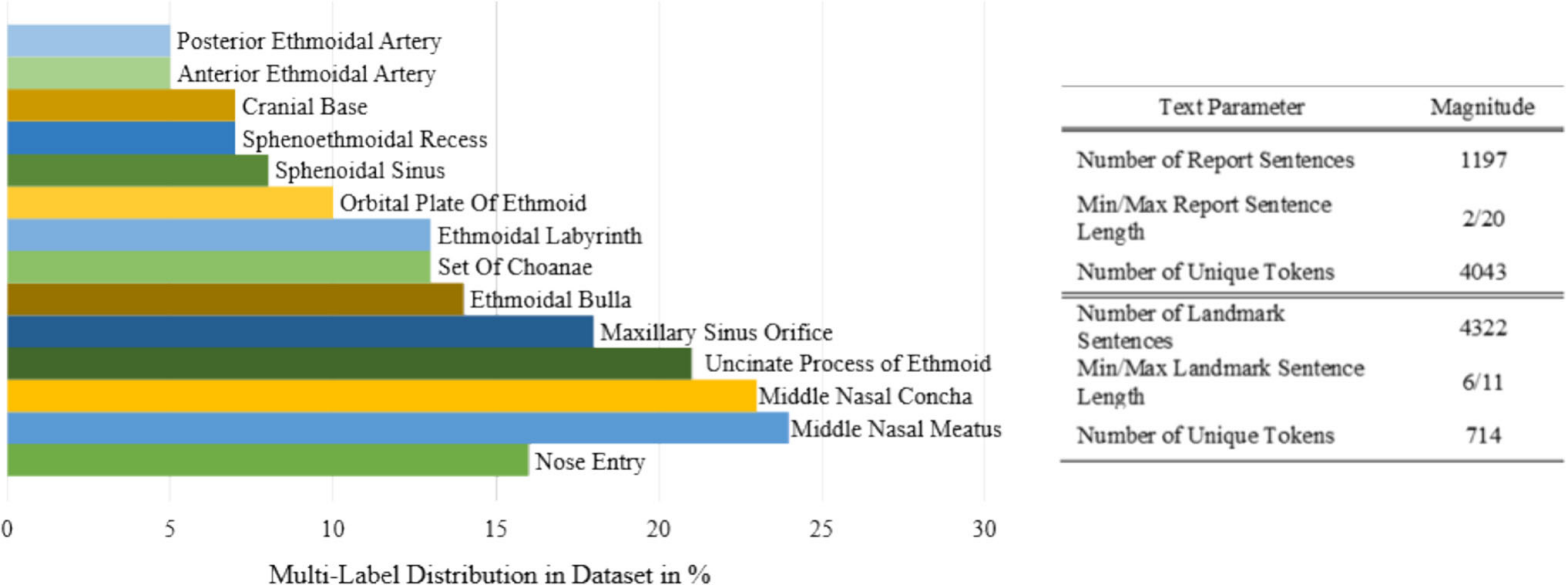
Overview of the label distribution across the sampled 130.000 image frame of the dataset (left) and text parameters for the structured and natural language datasets (right). Distribution percentages exceed 100% as multiple labels can be present at the same time during observations

Our study relies on a single, fully synchronized dataset assembled from three previously independent studies. We first selected thirty video recordings of FESS that together span roughly sixteen hours. Using the sentence‑level annotation protocol introduced in our work on language‑based workflow translation [19], we segmented each recording into “activities”, contiguous time windows in which one anatomical configuration remained stable; this yielded 4 322 annotated activities. The resulting label distribution is markedly skewed. The middle nasal meatus, middle nasal concha and uncinate process each appear in more than 20% of all annotated frames, whereas the anterior and posterior ethmoidal arteries are present in fewer than 10%. Within every activity we sub‑sampled the video at five frames per second and, following our image classification study [20], assigned to each frame a 16‑dimensional binary vector indicating the presence of the middle nasal meatus, uncinate process, and fourteen additional landmarks. To add natural‑language context, we drew on the 1197‑sentence commentary corpus from our earlier report‑generation paper [13]. Three senior ENT surgeons retrospectively followed each procedure recording and marked the first and last frame in which the report sentence accurately described the intra‑operative scene and also assigned a distinct keyword for this step. For any video frame t we thus obtained (1) the binary landmark vector, (2) the natural‑language landmark description originating from the workflow annotation, and (3) the “current” report sentence together with its keyword as well as the cumulative report history up to t. Frames for which one of these channels was missing were discarded. The resulting dataset comprises approximately 130.000 frames, each paired with structured landmark labels and free‑text commentary. This alignment enables the three target tasks of our study at the same temporal resolution (Figs. 1, 2).

### Task definitions

#### Task 1: Image Classification

This task is a multi-label classification problem, where each endoscopic image is associated with one or more anatomical landmarks that can be observed. It can also be interpreted as a form of image captioning of the endoscopic movement in situ. Endoscopic images were annotated with defined labels from [12]. Each annotation contained an arbitrary combination of 16 prominent landmarks that appeared alone or in combination during annotation. 

The task was then to classify the multiple labels that are present. Let ${f}_{C} :I \times T \to C$ be the classification function that takes images $I$ of a recorded video at a specific time T to produce classifications $C$ with $C=\{c\left({i}_{1},{t}_{1}\right),c\left({{i}_{2},t}_{2}\right),\dots |{t}_{n}\in T,{i}_{m}\in I\}$ where $c(i,t)$ are individual class vectors. Using the approach in [13], we construct an ordered set of labels $L$ of our annotated landmarks with$L=\{{l}_{1},{l}_{2}, \dots , {l}_{16}$}. The class vector $c(i,t)$ is then constructed with binary encoding as.

$$c\left(i,t\right)=[{b}_{1},{b}_{2}, \dots , {b}_{16}],$$

Where

$${b}_{j}=\left\{\begin{array}{c}1, if {l}_{j} is present in i(t)\\ 0, otherwise\end{array}\right.$$

A classifier would then be optimized to output a candidate prediction $\widehat{c}(i,t)$ that minimizes the average binary cross-entropy loss:

$${\mathcal{L}}_{\mathrm{BCE}}\left(c,\widehat{c}\right)=\frac{1}{16}\sum_{j=1}^{16}-({c}_{j}\mathrm{log}{\widehat{c}}_{j}+\left(1-{c}_{j}\right)\mathrm{log}(1-{\widehat{c}}_{j})$$

where ${c}_{j}$ and ${\widehat{c}}_{j}$ are the ${j}^{\mathrm{th}}$ component of the target and prediction vectors. The loss calculation is performed in the same way as image segmentation is done but with only 16 pixels to process.

#### Task 2: Landmark Prediction

This task was introduced to predict future anatomical landmarks from annotated videos of ENT endoscopic surgeries. Endoscopic videos were annotated where a specific landmark or combination of landmarks was observed. Using the annotation scheme in [12], each activity corresponds to video segment of arbitrary duration and has a sentence-level description ${S}_{D}$ in the structure:

$${S}_{D}= \left(Step Count, Main Cavity, Landmark Group, Landmarks , Movement Direction\right)$$

To make use of the embeddings from the text encoder, we translated the used taxonomy into a natural language form, e.g., turning the concept *concha_nasalis_media_group* into *“group of the medial nasal concha”* or *Uncinate_process_of_ethmoid into “Processus uncinatus”*. The task is then to predict from a current natural language description where the endoscope will most likely move toward. We chose a sequence-to-sequence style approach and constructed description pairs $\{{(s}_{{D}_{1}},{s}_{{D}_{2}}),{(s}_{{D}_{2}},{s}_{{D}_{3}}),\dots \}$. This concept is based on work about transfer learning of text-to-text models like T5 [14]. The model produces description words by maximizing the conditional probability:

$${s}_{D}=\left[{w}_{1},{w}_{2}, \dots , {w}_{n}\right]$$ and $${w}_{j+1}=argmax \prod_{j=0}^{n}p\left({\widehat{w}}_{j+1}|{w}_{j}\right)$$

with ${w}_{j}$ being the ${j}^{\mathrm{th}}$ word in the description sentence and $p\left({\widehat{w}}_{j+1}|{w}_{j}\right)$ being a probability for a candidate word ${\widehat{w}}_{j+1}$ of the final selected description word ${w}_{j+1}$. This auto-regressive word-by-word behavior is essentially a probability distribution of word relations.

The current procedure duration $t$ and the current observation duration were added as decimal numerals to address data synchronization. With the time-dependency, we can specify a prediction function ${f}_{P} :{S}_{D}\times T \to {S}_{D}$ with

$${s}_{D}\left(t+1\right)=argmax \prod_{j=0}^{m}p\left({\widehat{w}}_{m}(t+1)|{w}_{m-1}(t+1)\right)\text{ and}$$

$${s}_{D}\left(t+1\right)={f}_{P}\left({s}_{D}\left(t\right)\right)= {f}_{P}\left(argmax \prod_{j=0}^{n}p\left({\widehat{w}}_{n}(t)|{w}_{n-1}(t)\right)\right).$$

Here, ${\widehat{w}}_{n}\left(t\right)$ and ${\widehat{w}}_{m}(t+1)$ depict the conditional probability of having the complete descriptions ${s}_{D}\left(t\right)$ and ${s}_{D}\left(t+1\right)$. 

The text decoder in our VLM produces the words of ${s}_{D}\left(t+1\right)$ in succession as described above with a starting token and based on the embedding of ${s}_{D}\left(t\right)$ through a text encoder. The model is optimized on this prediction function using the KL divergence loss:

$${\mathcal{L}}_{\mathrm{KLD}}\left({P}_{{S}_{D}}||{Q}_{{S}_{D}}\right)={\sum }_{i=1}^{n}{P}_{{S}_{D}}\left({w}_{n}|{w}_{1},\dots ,{w}_{n-1}\right)log\frac{{P}_{{S}_{D}}\left({w}_{n}|{w}_{1},\dots ,{w}_{n-1}\right)}{{Q}_{{S}_{D}}\left({w}_{n}|{w}_{1},\dots ,{w}_{n-1}\right)}$$

Here, ${w}_{1}, \dots , {w}_{n}$ is the predicted sequence of words of the decoder model, ${P}_{{S}_{D}}(w)$ and ${Q}_{{S}_{D}}(w)$ are the ground truth and predicted conditional probabilities or negative log-likelihoods of the word sequence. The loss will be lower when the negative log-likelihood of ${Q}_{{S}_{D}}(w)$ for each predicted word resembles that of ${P}_{{S}_{D}}(w)$.

#### Task 3: Report Generation

This task assumes a language model can generate text sections of a surgical report based on spoken keywords during surgery. In this way, a keyword is combined with a partial report as the context window and parsed into an encoder-decoder model to output the next report sentence [13]. Let a dataset contain report sentences ${S}_{R}$ of a surgical report in the form of natural language text. For our task then exists a text generation function ${f}_{G} :H\times K\times T \to {S}_{R}$ than can procedure surgical sentences ${S}_{R}$ at times $T$ with keywords $K$ and the available context $H$ such that:

$${s}_{R}(t)={{f}_{G}(k}_{t},{h}_{t}|{k}_{t}\in K, {h}_{t}\in H,t\in T)$$

Here, a surgical sentence ${s}_{R}\left(t\right)$ at the duration $t$ of a procedure is generated with uttered keywords ${k}_{t}$ and the accumulated context ${h}_{t}$ with

$${h}_{t}=\{\left({k}_{1},{S}_{{R}_{1}}\right),\left({k}_{2},{S}_{{R}_{2}}\right),\dots ,({k}_{t-1},{S}_{{R}_{t-1}})$$

The context ${h}_{t}$ can be understood as a set of ordered pairs of keywords and report sentences of all previous timesteps up to $t-1$ in a procedure. Both ${k}_{t}$ and ${h}_{t}$ are used together as one string sequence as part of the text encoder input. During training, we use a fixed context window length of the 250 most recent tokens. We optimize a model for this task using a variation of cosine similarity loss with focal penalization of high similarity between the target and candidate sentences ${s}_{R}\left(t\right)$ and ${\widehat{s}}_{R}\left(t\right)$ with

$${\mathcal{L}}_{\mathrm{FCS}}\left({s}_{R},{\widehat{s}}_{R}\right)={\left(1- \frac{e\left({\widehat{s}}_{R}\left(t\right)\right)\cdot e({s}_{R}\left(t\right))}{\left|e\left({\widehat{s}}_{R}(t)\right)\right|\left|e({s}_{R}\left(t\right))\right|}\right) }^{\gamma }$$

Here, $e\left({\widehat{s}}_{R}\left(t\right)\right) \text{and }e({s}_{R}\left(t\right))$ represent the embedding vectors of the target and candidate sentences that we calculate with a pre-trained text encoder (see 3.4.1). The cosine similarity is penalized using $\gamma$ with values between 1 and 2. For our multi-task approach, we also use the keywords as the starting tokens at the beginning of the generation of ${\widehat{s}}_{R}\left(t\right)$.

## Vision-language model and trial architectures

### Model design

**Fig. 3:**
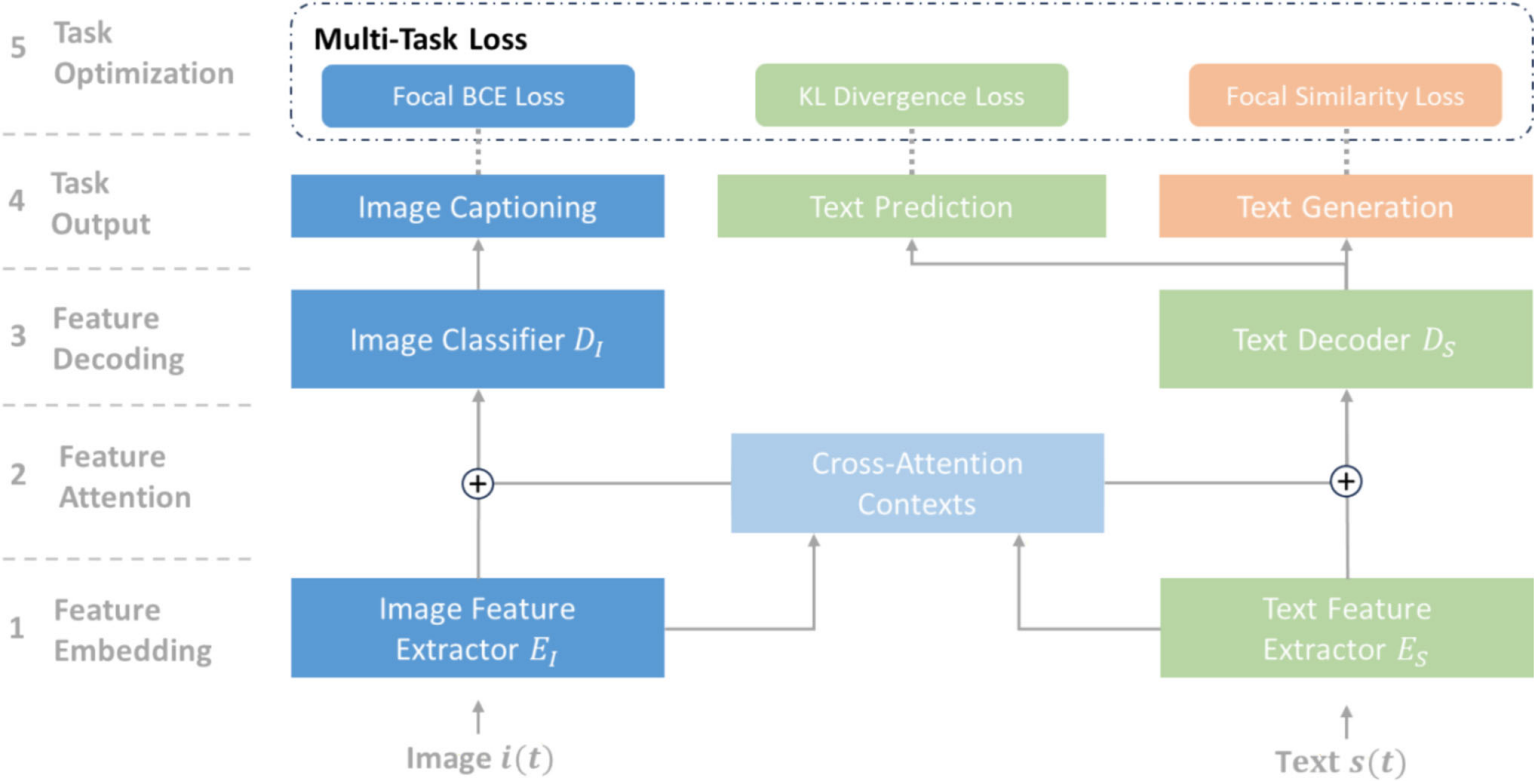
Overview of the VLM architecture using image and feature extractors to produce embeddings. Embedded features then pass a cross-attention layer, whose text and image contexts are concatenated with the respective embedding and passed into the text decoder and the image classifier to perform the downstream tasks

First, we use the encoders ${E}_{S}, {E}_{I}$ to produce text and image embeddings (Fig. 3). The embeddings are passed into a cross-attention layer, producing context vectors that are concatenated with each respective embedding. The concatenated vectors are then passed into the text decoder ${D}_{S}$ and the image classifiers ${D}_{I}$ to perform the downstream tasks. For each respective task, the model is optimized with a corresponding objective function (see Sect. 3.1). We implemented a model version using 61M parameters (“small”) with lightweight encoders and a base version with 176M parameters (“base”) (see Table 1). Where possible, pre-trained encoders are used to produce more selective embeddings for medical texts and endoscopic videos. Additionally, a pre-trained CLIP model was used without fine-tuning as a baseline.

**Table 1:** Encoder and decoder selection for the proposed VLM architecture and the baseline CLIP model. Encoder and decoder sizes are rounded numbers of the model parameters in millions that are optimized during training

VLM archiecure	VLM small	VLM base	CLIP [15]
Image encoder model	EfficientNetV2^1^	ENDO-FM (ViT B16) [16]	ViT-B/16^2^
Image encoder pre-training	SimCLR [17]/25k images	–	–
Image encoder size	**24M**	**88M**	**88M**
Text encoder model	T5-Efficient-Mini^3^	DistilRoBERTa-Base	Transformer
Text encoder pre-training	Bag-of-Words/4M tokens	Bag-of-Words/4M tokens	–
Text encoder size	**31M**	**82M**	**82M**
Image decoder model	MLP (3-Layer FC)	MLP (3-Layer FC)	MLP (1-Layer FC)
Image decoder size	**3M**	**3M**	**3M**
Text decoder model	2-Layer BiLSTM	2-Layer BiLSTM	2-Layer BiLSTM
Text decoder size	**3M**	**3M**	**3M**
VLM model size	**61M**	**176M**	**176M**

Bold values signify the best results

^1^https://huggingface.co/timm/tf_efficientnetv2_b3.in21k

^2^https://huggingface.co/google/vit-base-patch16-224

^3^https://huggingface.co/google/t5-efficient-mini

### Training and objective

We adopt a single-pass parameter update for all our multi-task models in which every parameter update is driven by a joint forward–backward pass that includes all three tasks. Each training mini-batch is constructed as the concatenation of three equal-sized sub-batches (Task 1 images, Task 2 image + description pairs, Task 3 image + keyword + report context). During the forward pass, the shared vision encoder processes the image portion of every sample, while the text encoder processes the corresponding textual inputs. The decoder heads then generate the multi-label landmark logits, the next structured landmark sentence, and the next free-text report sentence. All experiments reported in the main text use the geometric‐mean multi-task objective [18]

$${\mathcal{L}}_{\mathrm{GL}}= \sqrt[3]{{\mathcal{L}}_{\mathrm{BCE}}{\mathcal{L}}_{\mathrm{KLD}}{\mathcal{L}}_{\mathrm{FCS}}}$$

where ${\mathcal{L}}_{\mathrm{BCE}}$ is a binary cross-entropy loss for landmark classification, ${\mathcal{L}}_{\mathrm{KLD}}$ is a KL divergence loss for structured-text prediction, and ${\mathcal{L}}_{\mathrm{FCS}}$ is cosine-similarity focal loss for report generation. This scale-invariant formulation balances the three tasks without manual weighting. Alternative losses (focused-image, focused-text, simple average) were evaluated for completeness and are reported in section "Ablation studies".

We use a 90/10-split ratio for the training and validation data. All encoders have their weights frozen during downstream training and are not updated during back-propagation. For all training sessions, we employ the same batch size of 128 with the RADAM optimizer, a learning rate of l = 1e−4, and a warm-up over 10 epochs. As hardware two NVIDIA RTX A4500 with 20 GB of GPU memory are used. All training sessions are performed using PyTorch 1.9. With an average training over 100 epochs, one cross-validation session with single-pass training lasted around 6 h.

## Studies

### Cross-validation studies

We adopt a leave-one-patient-out (LOPO) protocol to ensure generalization to unseen subjects. In each of 30 folds one patient’s procedure videos serve exclusively as the test set. From the remaining 29 patients, we create an internal 90/10 split per patient (stratified by landmark class)—90% of frames joined the training pool, and 10% formed the validation pool for early stopping. Hence, the reported metrics are averaged across 30 independent folds, each trained on ~ 117 k frames and validated on ~ 13 k frames, while the held-out patient’s ~ 4 k frames were never seen during training or hyper-parameter tuning. Task results for the two trial architectures are compared with the results from our previous works using established vision-language metrics. All presented values are averaged over the cross-validation outputs. Ablation results are provided for individual tasks as well as the loss function strategy on the VLM base model. Semi-quantitative findings were accumulated for positive and negative multi-task results of the VLM base model, where positive results were defined as having a mean *f*1-score of over 0.65 and a correct landmark classification. A negative result has an incorrect landmark classification and a mean *f*1-score under 0.3. We provide examples for text and image attention values for good and bad results.

### Ablation studies

#### Cross-modal influence

To isolate the contribution of cross-modal information, we trained four model variants: (A) our full VLM Base model, (B) an image-only model (vision encoder + classifier; language decoder removed), (C) a text-only model (language decoder conditioned only on text; vision encoder frozen and ignored), and (D) a VLM without cross-attention (image and text streams processed independently). Table 4 summarises the outcomes averaged over the leave-one-patient-out folds.

#### Variation of loss strategy

Multi‑task optimization is dependent on how the individual task losses are combined. If one loss dominates, the shared encoders may overfit that task and under‑represent the others. We, therefore, compared the following loss strategies:

1. A geometric loss (GL) that equalizes relative progress across tasks and is scale‑invariant.
2. An Image‑focused loss (FL‑img) deliberately tilts the gradient budget toward landmark classification to test whether emphasising the vision branch benefits, or harms, the remaining language tasks.
3. A Text‑focused loss (FL‑txt) that mirrors this idea for the two language objectives, probing whether extra weight on textual signals can lift linguistic quality without degrading spatial accuracy.
4. An arithmetic loss (AL) provides a naïve, equal‑weight baseline, useful for detecting whether more sophisticated weighting actually matters.

By using these four strategies we can determine whether balanced weighting, task‑specific emphasis or simple averaging yields the most stable cross‑modal performance. We define the focused loss (FL) strategies for image and text tasks as:

$${\mathcal{L}}_{\mathrm{FL},\mathrm{Image}}={\mathcal{L}}_{\mathrm{BCE}}\sqrt{{\mathcal{L}}_{\mathrm{KLD}}{\mathcal{L}}_{\mathrm{FCS}}}$$ and $${\mathcal{L}}_{\mathrm{FL},\mathrm{Text}}=\break {\mathcal{L}}_{\mathrm{KLD}}{\mathcal{L}}_{\mathrm{FCS}}\sqrt{{\mathcal{L}}_{\mathrm{BCE}}}$$

And we define the average loss strategy (AL) as:

$${\mathcal{L}}_{\mathrm{AL}}= \frac{{\mathcal{L}}_{\mathrm{BCE}}+{\mathcal{L}}_{\mathrm{KLD}}+{\mathcal{L}}_{\mathrm{FCS}}}{3}$$

## Results

Unless otherwise noted, ‘baseline’ refers to the ResNet‑50 landmark classifier, the transformer-based structured‑text predictor, and the Bi‑LSTM report generator reported in Bieck et al. [13, 19, 20].

### Cross-validation studies

The VLM base raises landmark‑classification *f*1 from 0.47 to 0.59 (+ 12%) and boosts report‑generation *f*1 from 0.50 to 0.64 (+ 14%). The structured text prediction, by contrast, gains little precision improvements, but does not reach the single‑task transformer, suggesting that highly templated sentences leave limited room for improvement. Benchmarking against three external vision‑language baselines, EndoVit with its vision‑heavy backbone passes CLIP on landmark classification but lags behind our VLM base in text metrics. SurgicalGPT shows the opposite trend, its language‑centric design reaches a higher text generation *f*1 shortly after our VLM base, yet loses out on EndoVit on landmark classification (Table 2).

**Table 2:** Results for single-task and multi-task model comparison (no ablation, all metrics are averaged over 30 LOPO folds, f-1—*F*1 score, pr—precision, re—recall, bl2—BLEU2, rg-1—ROUGE-1, coss—cosine similarity, f-1*—adopted *F*1-score from BLEU2 and ROUGE1)

Model	Model size	Landmark classification			Text prediction			Text generation			
		f-1	pr	re	f-1	pr	re	f-1*	bl2	rg-l	coss
Baseline—transformer [19]	15M	–	–	–	**0.70**	0.67	**0.75**	–	–	–	–
Baseline—S2S/Bi-LSTM [13]	8M	–	–	–	–	–	–	0.50	0.60	0.43	0.64
Baseline—ResNet50 [20]	25M	0.47	0.54	0.43	–	–	–	–	–	–	–
VLM small (ours)	61M	0.52	0.55	0.50	0.65	0.68	0.64	0.53	0.62	0.47	0.65
VLM base (ours)	176M	**0.59**	**0.62**	**0.58**	0.68	**0.69**	0.68	**0.64**	**0.67**	**0.62**	**0.70**
CLIP	176M	0.52	0.55	0.52	0.59	0.65	0.55	0.53	0.52	0.54	0.60
EndoViT [8]	132M	0.56	0.59	0.54	0.66	0.68	0.66	0.60	0.63	0.58	0.66
SurgicalGPT [7]	**320M**	0.55	0.58	0.54	0.67	0.67	0.67	0.63	0.66	0.60	0.68

Bold values signify the best results

Table 3 reports the precision, recall and *F*1 for the landmark-specific classification results using the VLM base model. The classification performance correlates strongly with the class frequency, where high-prevalence classes such as middle nasal meatus (24% occurrence in multi-labels) achieve the highest *f*1 scores above *f*1 = 0.70, whereas rare classes such as Cranial Base (5%) only reach an *f*1 = 0.50. The anterior and posterior ethmoidal arteries, each with the lowest occurrence in the dataset, yield the lowest scores.

**Table 3:** Lable-wise performance for classification task supported by cross-attention with text embedding vor the VLM Base model (all metrics are averaged over 30 LOPO folds, f-1—*F*1 score, pr—precision, re—recall)

Landmark label	Occurance in dataset	Classification performance		
	%	f-1	pr	re
Nose entry	16	0.68	0.70	0.66
Middle nasal meatus	24	0.75	0.78	0.79
Middle nasal concha	23	0.72	0.74	0.70
Uncinate process of ethmoid	21	0.70	0.73	0.68
Maxillary sinus orifice	18	0.68	0.70	0.66
Ethmoidal bulla	14	0.66	0.68	0.64
Set of choanae	13	0.62	0.64	0.6
Ethmoidal labyrinth	13	0.60	0.62	0.58
Orbital plate of ethmoid	10	0.57	0.59	0.55
Sphenoidal sinus	8	0.52	0.54	0.51
Sphenoethmoidal recess	7	0.50	0.52	0.49
Cranial base	7	0.50	0.52	0.48
Anterior ethmoidal artery	5	0.40	0.42	0.38
Posterior ethmoidal artery	5	0.38	0.40	0.36
Macro average overall	–	0.59	0.62	0.58

### Ablation studies

The variant A with a full VLM and cross‑attention achieved the strongest performance across all tasks. Removing the language pathway in variant B reduced landmark *f*1 to 0.55 (− 0.04) and, as expected, produced no text outputs. Removing the vision pathway in variant C reduces the structured‑text prediction slightly but lowers free‑text metrics noticeably to an *f*1 of 0.61. The last variant D without cross-attention shows intermediate *f*1 scores 2–5% below the VLM base with cross-attention (Table 4).

**Table 4:** Ablation study on contribution of modalities (all metrics are averaged over 30 LOPO folds, f-1—*F*1 score, pr—precision, re—recall, bl2—BLEU2, rg-1—ROUGE-1, coss—cosine similarity, f-1*—adopted *F*1-score from BLEU2 and ROUGE1)

Model	Model size	Landmark classification			Text prediction			Text generation			
		f-1	pr	re	f-1	pr	re	f-1*	bl2	rg-l	coss
A-VLM base	176M	**0.59**	**0.62**	**0.58**	**0.68**	**0.69**	**0.68**	**0.64**	**0.67**	**0.62**	**0.70**
B-Image-only base	88M	0.55	0.57	0.54	–	–	–	–	–	–	–
C-text-only	88M	–	–	–	0.65	0.66	0.64	0.59	0.61	0.58	0.60
D-VLM w/o cross-att	176M	0.57	0.56	0.58	0.66	0.67	0.67	0.61	0.62	0.59	0.62

Bold values signify the best results

The geometric mean (GL) strategy attains the highest scores. Shifting the weight toward the image task (IMG‑FL) reduces landmark classification *f*1 to 0.57 (− 0.02) and lowers report generation *f*1 to 0.62 (− 0.02), while leaving the structured text prediction mostly unchanged. Favoring the text tasks (TXT‑FL) preserves language performance but also drops landmark classification *f*1 to 0.57 (− 0.02). A simple arithmetic average of the three losses (AL) matches the geometric mean on the classification *f*1 yet is lower on every language metric.

## Discussion

Our study demonstrates the feasibility of integrating vision-language models for multi-task assistance, shifting from traditional vision-only approaches. By leveraging image and text embeddings, we achieved notable performance gains in classification and report generation tasks. Linking separated image and text datasets required extensive manual annotation, showing a need for more streamlined and automated annotation. Future efforts should prioritize developing improved annotation schemes and better integration of surgeon commentary and report documentation. The quality of dataset classes remains challenging, as intrinsic class ambiguity impacts model overfitting. The task results indicate a notable improvement when models could associate image and text embeddings for classification and text generation tasks. The prediction task, which focused on structured text-to-text transfer learning, demonstrated limited improvement. This suggests that structured text does not benefit from transformers fine-tuned to natural language data. Negative transfer effects between tasks highlight the importance of carefully balancing multi-task learning objectives. The benchmarking results show that both EndoVit and SurgicalGPT compare reasonably well but sit between the CLIP model and our domain-specific VLM. This suggests that multi-task optimisation with ENT‑biased encoders yields a measurable margin over strong general‑purpose systems while preserving model compactness and deployment efficiency. 

**Fig. 4:**
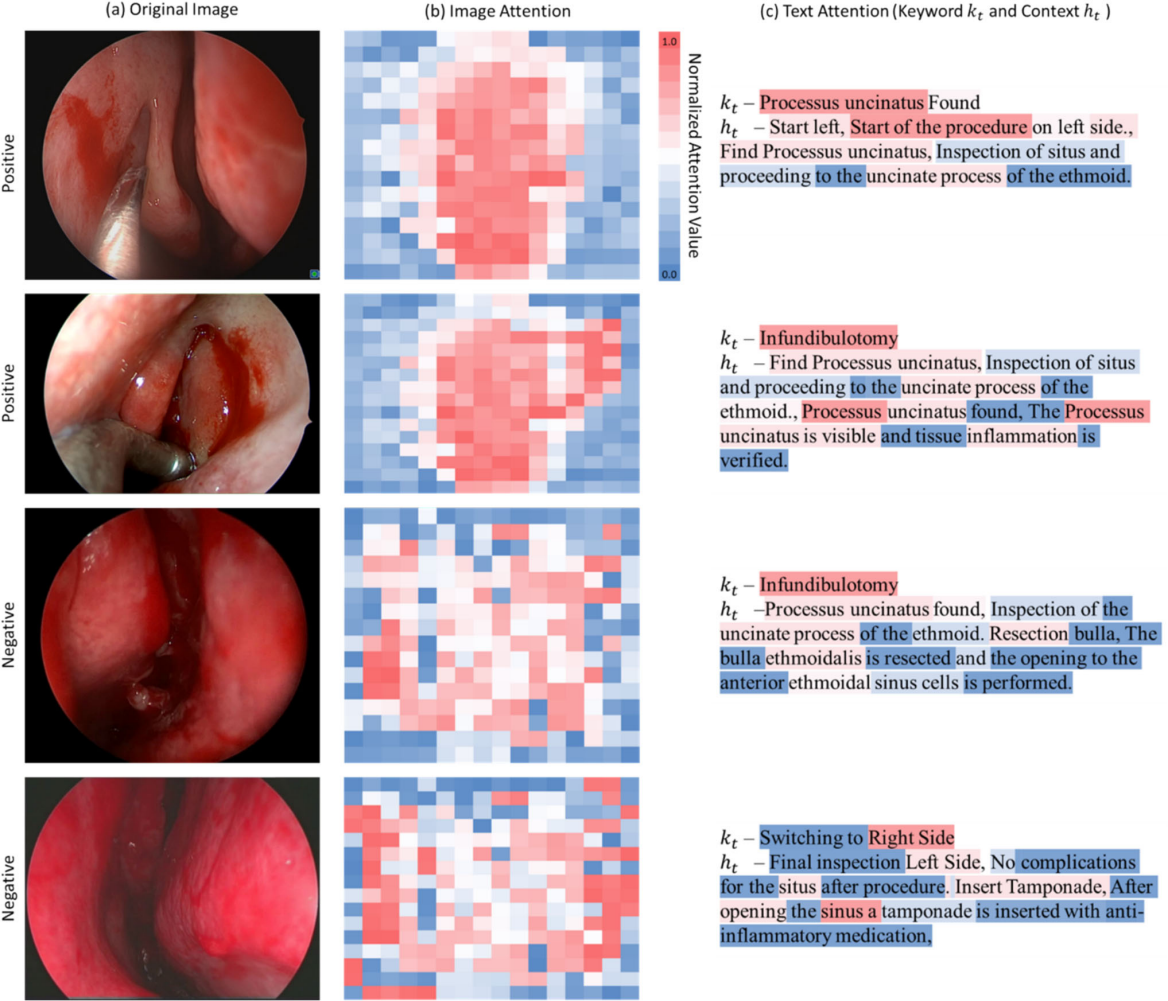
Positive and negative examples for multi-task results. Original images (**a**) Attention values for images (**b**) and text passages (**c**) indicate the assigned weight during feature embedding and may correlate with relevance for multi-task results. A stronger red marking indictes higher weighting, a stronger blue coloring indicates less weight

The class-wise analysis in Table 5 confirms a pronounced long-tail effect in Task 1. The strong correlation between class frequency and *f*1 underlines the need for targeted strategies against class imbalance. Incorporating uncertainty measures into the decision layer may reduce the model’s tendency to omit rare landmarks. Addressing these issues should further improve overall robustness without compromising the high accuracy already achieved on dominant anatomical structures (Fig. 4).

**Table 5:** Ablation study on the variation of loss strategies (GL—geometric loss, IMG FL—focused loss on image task, TXT FL—focused loss on text tasks, AL—averaged loss)

Model	Landmark classification			Text prediction			Text generation			
	f-1	pr	re	f-1	pr	re	f-1*	bl2	rg-l	coss
VLM base + GL	**0.59**	**0.62**	**0.58**	**0.68**	**0.69**	**0.68**	0.64	0.67	0.62	**0.70**
VLM base + IMG FL	0.57	0.61	0.55	0.67	0.68	0.67	0.62	0.67	0.59	0.65
VLM base + TXT FL	0.57	0.60	0.55	0.67	0.67	0.67	**0.65**	**0.67**	**0.64**	0.69
VLM base + AL	0.59	0.62	0.57	0.66	0.67	0.66	0.61	0.65	0.58	0.64

Bold values signify the best results

The ablation study suggests that visual input adds meaningful scene context into the language decoder for free-text generation, while text embeddings seem to contribute little to the inherently vision-dominant landmark task. Moreover, simply concatenating independent image and text features cannot substitute for explicit cross-attention. These findings suggest that future work should prioritize visual-language coupling when the objective is narrative output, whereas vision-only models may remain adequate for image classification tasks. The geometric-mean objective outperforms the other schemes because it balances gradients in a scale-invariant way, preventing any single task from dominating optimisation. In contrast, the focused losses tilt the gradient budget toward a chosen modality. The arithmetic average appears fair but is still sensitive to absolute loss magnitudes, which explains its weaker language metrics. These findings suggest that multi-task ENT models benefit from loss functions that equalize relative task progress. In future work, exploring dynamic weighting strategies that adapt to task difficulty over training time should be investigated. The current VLM approach does not explicitly account for long-term spatio-temporal relationships, which could enhance the robustness of feature embeddings and temporal coherence as highlighted by [21]. New strategies are needed to address these limitations, potentially by redefining task goals or incorporating additional contextual features.

## Conclusion

We present a single, compact vision‑language model that unifies three assistance functions for sinus surgery: landmark recognition, next‑step anatomical description, and operative report generation. By adapting an established encoder–decoder backbone with endoscopy‑specific image and text encoders, the model improves task performance up to 14%. It is comparable with larger models, such as SurgicalGPT, yet remains deployable on standard OR hardware. Our ablations show that visual features are beneficial for text-based tasks and that cross‑attention is essential for effective fusion of image-text embeddings. Overall, the approach for vision-language in endoscopic assistance is promising and the influence of including natural language data from narrations of domain experts could potentially open up more promising model applications.
